# Gender-specific symptom outcomes on cariprazine treatment: a 12-month naturalistic longitudinal follow-up study in schizophrenia

**DOI:** 10.3389/fpsyt.2026.1795351

**Published:** 2026-06-23

**Authors:** Claudia Carmassi, Virginia Pedrinelli, Andrea Bordacchini, Berenice Rimoldi, Livia Parrini, Carlo A. Bertelloni, Valerio Dell’Oste

**Affiliations:** 1 Department of Clinical and Experimental Medicine, University of Pisa, Pisa, Italy; 2 Department of Mental Health and Addiction, Azienda USL Toscana Nord-Ovest, Livorno, Italy; 3 Department of Mental Health and Addiction, Azienda USL Toscana Nord-Ovest, Massa, Italy; 4 Department of Mental Health and Addiction, Azienda USL Toscana Nord-Ovest, Lucca, Italy

**Keywords:** cariprazine, psychosis, schizophrenia, sex difference, treatment response, women

## Abstract

**Introduction:**

A growing body of literature is focusing on third-generation antipsychotics and their unique characteristics, but few studies have examined gender as a crucial factor in response profiles. The present study aims to address this gap by analyzing the outcomes of 12-month naturalistic treatment with cariprazine to elucidate changes in specific psychopathological domains between men and women.

**Methods:**

The present 12-month longitudinal naturalistic study involved a sample of individuals diagnosed with schizophrenia according to the DSM-5-TR treated with cariprazine at the outpatients’ psychiatric services of a major university and community hospitals in Italy. The assessments conducted included sociodemographic data, the Structured Clinical Interview for the DSM-5 (SCID-5), and the Positive and Negative Symptom Scale (PANSS) Total and Subscale scores, as well as the PANSS-derived Marder factors. The PANSS was administered at three time points: before starting the treatment with cariprazine (T0), after 6 months (T1), and after 12 months (T2).

**Results:**

Fifteen male and 17 female subjects were assessed at the three time points. The mean dose of cariprazine was 4.2 ± 1.3 mg for men and 4.0 ± 1.5 mg for women. Both genders exhibited improvements in all PANSS subscale symptoms after 6 and 12 months of cariprazine treatment compared to the baseline, with the only exception of the Uncontrolled hostility/excitement Marder factor among men. Progressive improvements through time points in symptom subsca\les were found in both sexes, reaching numerical differences in every PANSS subscale in both sexes at T2. Gender specifc response profiles emerged after 6 and 12 months of treatment in the PANSS subscales and items in men and women.

**Discussion:**

Cariprazine exhibited significant efficacy in both sexes, with no significant differences between men and women despite a gender specific response profile emerged. Additional studies are needed to further investigate the efficacy profile and long-term outcome of cariprazine treatment by gender.

## 1 Introduction

Increasing evidence suggests the relevance of a gender-specific approach to the clinical characterization of mental disorders to define personalized and more effective treatments. The literature on patients with schizophrenia suggests that clinical features, course of illness and prognosis, peak of incidence, and responsiveness to treatment may vary widely between men and women (1, 2). While the highest incidence rate has been reported to occur approximately in the mid-20s among men, it has often been described as characterized by two peaks among women, with the first occurring in the late-20s and the second during the postmenopausal age (3, 4). Regarding symptoms, men are more often associated with greater negative symptoms and more severe substance use, a higher risk of developing cognitive impairment, and worse outcomes in work and social functioning. On the other hand, women are more frequently associated with depressive symptoms and positive symptoms of psychosis (5). Furthermore, premorbid trajectory has been distinguished by a more frequent presence of mood disorders among women and a greater tendency for the development of substance use disorder among men (5).

Many of the differences ascribed to gender have been attributed to the so-called protective theory of estrogen hormones (6–8), indicating that women are somewhat protected from the onset of symptoms or from a higher severity of symptoms until menopause. Estrogen levels have also been linked in the literature to the worsening of psychotic symptoms during the premenstrual period (9).

Different studies have also examined the difference in pharmacological response between the two sexes. Regarding antidepressants, differences in neurotransmitter metabolism (10), as well as variations in hormonal, physiological, or pharmacodynamic (11) factors, that may contribute to different outcomes have been highlighted (12, 13). Gender-specific differences in the response to antipsychotics and the side effects reported have also been noticed, with studies highlighting how women may comparably respond to lower doses of antipsychotic medication but may also experience more side effects (14, 15).

Literature data seem to suggest the fact that women tend to report different patterns of treatment response to antipsychotic treatment. A recent meta-analysis looking at 22 registration trials for antipsychotic drugs highlighted that the response to antipsychotic treatment was greater in women, with a lower number of patients needed to treat to achieve comparable results to men, and that treatment efficacy was independent of initial negative symptom severity and menopausal status, although it decreased with age (16). Furthermore, increasing data suggest that the peculiarity of the presentation of psychosis in both genders could lengthen the time needed to reach the diagnosis of schizophrenia if not supported by adequate assessments; difficulties in differential diagnosis with affective disorders may also occur. The subsequent possibility of insufficient treatment may increase the time of untreated illness, which is directly related to greater disease-induced neurodegeneration and a worse long-term prognosis (17). Nevertheless, female sex is often underrepresented in many pharmacological studies, and this may not provide us with sufficiently accurate data for the use of antipsychotic drugs in the female population (18). Moreover, women are less represented in registration trials, particularly in specific phases of their lifespan (e.g., pregnancy) that are frequently challenging in clinical practice.

Dual antipsychotics have recently been introduced as an advanced treatment option, capable of targeting symptoms inadequately addressed by earlier treatments, such as impaired cognition, emotional bluntness, and social impairment. These new treatments have shown clinical responses and fewer side effects compared to first- and second-generation antipsychotics (19, 20). Among the so-called third-generation antipsychotics, cariprazine stands out due to its unique activity on the D2, 5-HT1A, 5-HT2A, 5-HT2B, and D3 receptors, which gives it the potential to be effective across a broad range of symptom domains (21). Our study aims to address this gap by evaluating the efficacy of cariprazine treatment in a balanced sample of men and women over a 12-month period and by highlighting sex differences in response through the administration of validated scales.

## 2 Materials and methods

### 2.1 Study sample

The present study included a total sample of 32 subjects with a DSM-5-TR diagnosis of schizophrenia enrolled at the adult outpatient psychiatric services of the Psychiatric Clinic of a major university hospital in central Italy (Azienda Ospedaliero-Universitaria Pisa, AOUP, Pisa, Italy) and at the Psychiatric Unit of the Mental Health Department of Massa (Azienda USL Toscana Nord Ovest, Massa, Italy), between January 2022 and January 2023, and naturalistically treated with cariprazine for 12 months. A detailed description of the study methods is reported elsewhere (22). The inclusion criteria comprised a DSM-5-TR diagnosis of schizophrenia and an age of at least 18 years old at the time of enrollment in the study. The exclusion criteria were insufficient knowledge of the Italian language or other difficulties to verbal communication and a concomitant diagnosis of cognitive impairment, seizures or other neurological disorders, and a history of traumatic brain injuries. Written informed consent was obtained from all participants after receiving an accurate description of the study. All data were collected anonymously. The study was conducted in accordance with the Declaration of Helsinki and was approved by the Ethics Committee of the Area Vasta Nord Ovest Toscana (Italy).

### 2.2 Assessment instruments

All subjects enrolled were assessed by trained psychiatrists or residents in psychiatry of the Psychiatric Clinic of the University of Pisa, Italy. Patients attended monthly follow-up visits for clinical and therapeutic monitoring and to ensure treatment adherence. The assessments included the following: a datasheet for sociodemographic and clinical characteristics (including gender, age, marital status, employment status, educational status, family history of psychiatric disorder, physical and psychiatric comorbidities, psychopharmacological treatment characteristics, and number of prior hospitalizations before starting and during cariprazine treatment); the Structured Clinical Interview for Mental Disorders (SCID-5), for the diagnosis of schizophrenia and any other mental disorders/comorbidities; and the Positive and Negative Symptom Scale (PANSS), to measure the psychotic spectrum’s symptom severity. Both assessment instruments were administered at baseline before starting the treatment with cariprazine (T0), and the PANSS was also repeated after 6 (T1) and 12 (T2) months of cariprazine treatment. The PANSS includes 30 items and assesses the severity of psychopathology in adults with schizophrenia. Each item is assigned a score from 1 to 7, with a minimum score of 30 and a maximum score of 210. The PANSS includes three subscales: *positive symptoms* (7 items), *negative symptoms* (7 items), and *general psychopathology* (16 items) (23). Further factor analysis studies, conducted to investigate whether the 30 symptoms cluster into specific dimensions that could underlie distinct processes in schizophrenia, identified five factors (known as PANSS Marder factors), namely, *positive*, *negative*, *cognitive/disorganization*, *depression/anxiety*, and *excitability/hostility* dimensions (24, 25).

### 2.3 Statistical analysis

All statistical analyses were performed using the IBM SPSS Statistics (Version 22), IBM inc. (2021). Continuous variables were reported as mean ± standard deviation (SD), whereas categorical variables were reported as percentages. All tests were two-tailed and a *p*-value <0.05 was considered statistically significant. *t*-tests for equality of means were computed to compare PANSS total score, positive, negative, and general psychopathology scales, as well as the five PANSS Marder factors (positive symptoms, negative symptoms, disorganized thought, hostility/excitement, anxiety/depression) in both sexes between baseline (T0) and 6 months of follow-up (T1) and between baseline (T0) and 12 months of follow-up (T2). Moreover, the paired *t*-test non-parametric Wilcoxon test was performed to compare each PANSS scale item between T0 and T1 and T0 and T2 in both sexes.

## 3 Results

The total sample included 32 subjects with a diagnosis of schizophrenia, consisting of 15 men (46.9%) and 17 women (53.1%), with a mean age of 41.6 ± 10.7 years (40.9 ± 10.2 and 42.1 ± 11.3 in men and women, respectively, *p* = 0.760). The sociodemographic and clinical characteristics of the sample, stratified by gender, are summarized in **Table 1**. In terms of sociodemographic characteristics, five men (33.3%) and three women (17.6%) were married or cohabiting, nine men (60.0%) and six women (35.3%) were employed, and eight men (53.3%) and eight women (47.1%) had a high school degree.

**Table 1:** Sociodemographic and clinical characteristics in the total sample (*N* = 32), stratified by gender (men, *N* = 15; women, *N* = 17).

Sociodemographic/clinical characteristics	Males *N* (%)	Females *N* (%)	*p*
Married/cohabiting	5 (33.3)	3 (17.6)	0.306
Employed	9 (60.0)	6 (35.3)	0.162
High school degree	8 (53.3)	8 (47.1)	0.723
Current medical comorbidities			
*Obesity*	3 (20.0)	3 (17.6)	0.865
*Dyslipidemia*	4 (26.7)	2 (11.8)	0.281
*Hypertension*	3 (20.0)	2 (11.8)	0.522
*Diabetes mellitus*	1 (6.7)	1 (5.9)	0.927
Family history of psychiatric disorder	9 (60.0)	11 (68.8)	0.611
Current psychiatric comorbidities			
*Anxiety disorder*	4 (26.7)	3 (17.6)	0.538
*Obsessive–compulsive disorder*	2 (13.3)	1 (5.9)	0.471
*Alcohol or substance use disorder*	5 (33.3)	6 (35.3)	0.907
*Alcohol use disorder*	3 (20.0)	1 (5.9)	0.228
*Cannabinoid use disorder*	2 (13.3)	1 (5.9)	0.471
*Stimulant use disorder*	3 (20.0)	5 (29.4)	0.539
*Opioid use disorder*	0 (0)	3 (17.6)	0.087
Hospitalization before starting cariprazine treatment	8 (53.3)	7 (41.2)	0.492
Hospitalization during cariprazine treatment	2 (13.3)	1 (5.9)	0.417
Mean age	40.9 ± 10.2	42.1 ± 11.3	0.760
Cariprazine dose	4.2 ± 1.3	4.0 ± 1.5	0.648

Examining in detail the clinical characteristics of the sample, four men (26.7%) and three women (17.6%) had a current diagnosis of an anxiety disorder and two men (13.3%) and one woman (5.9%) suffered from obsessive–compulsive disorder. Approximately one-third of all participants, including five men (33.3%) and six women (35.3%), reported a current alcohol or substance use disorder: three men (20.0%) and one woman (5.9%) had a current alcohol use disorder; two men (13.3%) and one woman (5.9%) had a current cannabinoid use disorder; three men (20.0%) and five women (29.4%) had a current stimulant use disorder, and three women (17.6%) had a current opioid use disorder (see **Table 1**). A total of 31 (96.9%) patients, comprising 15 men and 16 women, had a 6-month treatment duration with cariprazine, whereas data on cariprazine treatment at T2 were available for 29 patients, comprising 14 men and 15 women. Results also showed that two men (13.3%) and one woman (5.9%) reported at least one hospital admission for relapses of the disease during the time of treatment with cariprazine, while eight men (53.3%) and seven women (41.2%) reported at least one hospital admission before starting treatment with cariprazine. Overall, no statistically significant gender differences emerged when comparing psychopharmacological therapy-related characteristics in the sample.

Looking at the PANSS subscales and total scores and PANSS-derived Marder factor scores at T0, T1, and T2, there were no significant differences in mean scores between men and women. However, numerical differences were observed at times: women scored numerically higher than men in the PANSS Positive symptoms subscale at T0 and T1, while scores were comparable at T2. Negative symptoms subscale showed comparable mean scores at T0 and T2, but the score at T1 was numerically higher for women. The General psychopathology score was numerically higher for men at every time point. Total score was higher for women at T0 and T1 and lower than men at T2. See **Tables 2**, **3** for details.

**Table 2:** PANSS total and domain scores in men and women at T0 (baseline: 17 women and 15 men), T1 (6-month follow-up: 16 women and 15 men), and T2 (12-month follow-up: 15 women and 14 men).

PANSS scores		Males mean ± SD	Females mean ± SD	p
Positive symptoms scale	T0	19.9 ± 4.9	23.8 ± 6.2	0.057
	T1	17.3 ± 6.0	19.4 ± 5.9	0.331
	T2	16.4 ± 5.9	16.3 ± 5.3	0.991
Negative symptoms scale	T0	20.9 ± 6.0	20.2 ± 9.2	0.804
	T1	17.1 ± 5.2	19.0 ± 9.7	0.493
	T2	15.6 ± 5.0	15.9 ± 7.9	0.885
General psychopathology scale	T0	48.7 ± 11.8	45.8 ± 9.2	0.439
	T1	41.4 ± 11.1	39.4 ± 11.9	0.629
	T2	37.9 ± 9.9	34.0 ± 9.0	0.283
Total score	T0	89.5 ± 17.7	89.9 ± 20.2	0.959
	T1	75.8 ± 19.1	77.8 ± 25.4	0.806
	T2	69.8 ± 18.2	66.3 ± 20.2	0.627

**Table 3:** PANSS Marder factor mean scores in men and women at T0 (baseline: 17 women and 15 men), T1 (6-month follow-up: 16 women and 15 men), and T2 (12-month follow-up: 15 women and 14 men).

PANSS Marder factors		Males mean ± SD	Females mean ± SD	p
Positive scale	T0	25.7 ± 6.7	24.7 ± 6.3	0.678
	T1	21.5 ± 6.6	20.6 ± 6.6	0.724
	T2	19.5 ± 6.1	18.0 ± 5.5	0.493
Negative scale	T0	20.8 ± 4.8	19.7 ± 8.4	0.649
	T1	16.6 ± 4.7	18.4 ± 8.9	0.477
	T2	15.3 ± 4.5	15.5 ± 6.8	0.933
Disorganized thought	T0	18.8 ± 4.9	19.5 ± 6.4	0.722
	T1	16.7 ± 5.0	17.1 ± 6.8	0.880
	T2	16 ± 4.4	14.8 ± 6.0	0.546
Uncontrolled hostility/excitement	T0	10.3 ± 3.6	12.1 ± 3.4	0.140
	T1	9.5 ± 3.2	10.2 ± 3.0	0.561
	T2	8.9 ± 3.0	8.7 ± 2.9	0.861
Anxiety and depression	T0	14.0 ± 4.8	13.8 ± 2.2	0.898
	T1	11.5 ± 4.4	11.5 ± 3.3	0.981
	T2	10.1 ± 4.3	9.3 ± 2.5	0.536

Examining PANSS total and subscale scores, statistically significant improvement was shown across all subscales and total score in both men and women between T0 and T1 and T0 and T2 (**Tables 4A**, **B**).

**Table 4A:** PANSS domain mean scores between T0 and T1 in the total sample (31 subjects) [women (N = 16) and men (N = 15)] (paired sample statistics).

PANSS scores		Females mean ± SD	p	Males mean ± SD	p
Positive symptoms scale	T0	23.4 ± 6.2	0.010	19.9 ± 4.9	0.031
	T1	19.4 ± 5.9		17.3 ± 6.0	
Negative symptoms scale	T0	20.4 ± 9.5	0.003	20.9 ± 6.0	0.017
	T1	19.0 ± 9.7		17.1 ± 5.2	
General psychopathology scale	T0	46.1 ± 9.5	0.008	48.7 ± 11.8	0.005
	T1	39.4 ± 11.9		41.4 ± 11.1	
Total score	T0	89.8 ± 20.8	0.005	89.5 ± 17.7	0.007
	T1	77.8 ± 25.4		75.8 ± 19.1	

**Table 4B:** PANSS domain mean scores between T0 and T2 in the total sample (29 subjects) [women (N = 15) and men (N = 14)] (paired sample statistics).

PANSS scores		Females mean ± SD	p	Males mean ± SD	p
Positive symptoms scale	T0	23.5 ± 6.4	<0.001	19.9 ± 5.0	0.004
	T2	16.3 ± 5.3		16.4 ± 5.9	
Negative symptoms scale	T0	19.4 ± 8.9	<0.001	20.5 ± 6.0	0.003
	T2	15.9 ± 7.9		15.6 ± 5.0	
General psychopathology scale	T0	45.8 ± 9.7	<0.001	49.2 ± 12.1	0.001
	T2	34.0 ± 9.0		37.9 ± 9.9	
Total score	T0	88.7 ± 21.0	<0.001	89.6 ± 18.3	0.001
	T2	66.3 ± 20.2		69.8 ± 18.2	

When comparing the PANSS-derived Marder factors, all subscale scores showed statistically significant improvement at T1 in both sexes apart from Uncontrolled hostility/excitement in men, which did not reach statistical significance at this time point. This trend was confirmed at T2, when statistically significant improvement was recorded in all subscales and in both genders except for Uncontrolled hostility/excitement in men. See **Tables 5A**, **B** for details.

**Table 5A:** Comparison of PANSS Marder factor mean scores between T0 and T1 in the total sample (31 subjects) [women (N = 16) and men (N = 15)] (paired sample statistics).

PANSS Marder factors		Females mean ± SD	p	Males mean ± SD	p
Positive scale	T0	24.5 ± 6.4	0.015	25.7 ± 6.7	0.003
	T1	20.6 ± 6.6		21.5 ± 6.6	
Negative scale	T0	20.0 ± 8.6	0.006	20.8 ± 4.8	0.008
	T1	18.4 ± 8.9		16.6 ± 4.7	
Disorganized thought	T0	19.2 ± 6.4	0.016	18.8 ± 4.9	0.024
	T1	17.1 ± 6.8		16.7 ± 5.0	
Uncontrolled hostility/excitement	T0	12.2 ± 3.4	0.031	10.3 ± 3.6	0.274
	T1	10.2 ± 3.0		9.5 ± 3.2	
Anxiety and depression	T0	13.9 ± 2.2	0.004	14.0 ± 4.8	0.005
	T1	11.5 ± 3.3		11.5 ± 4.4	

**Table 5B:** Comparison of PANSS Marder factor mean scores between T0 and T2 in the total sample (29 subjects) [women (N = 15) and men (N = 14)] (paired sample statistics).

PANSS Marder factors		Females mean ± SD	p	Males mean ± SD	p
Positive scale	T0	24.5 ± 6.6	<0.001	25.9 ± 6.9	<0.001
	T2	18.0 ± 5.5		19.5 ± 6.1	
Negative scale	T0	19.1 ± 8.1	<0.001	20.4 ± 4.7	0.002
	T2	15.5 ± 6.8		15.3 ± 4.5	
Disorganized thought	T0	18.7 ± 6.4	0.002	18.9 ± 5.0	0.007
	T2	14.8 ± 6.0		16.0 ± 4.4	
Uncontrolled hostility/excitement	T0	12.3 ± 3.5	0.002	10.4 ± 3.6	0.092
	T2	8.7 ± 2.9		8.9 ± 3.0	
Anxiety and depression	T0	14.0 ± 2.3	<0.001	13.9 ± 5.0	0.001
	T2	9.3 ± 2.5		10.1 ± 4.3	

**Figure 1:**
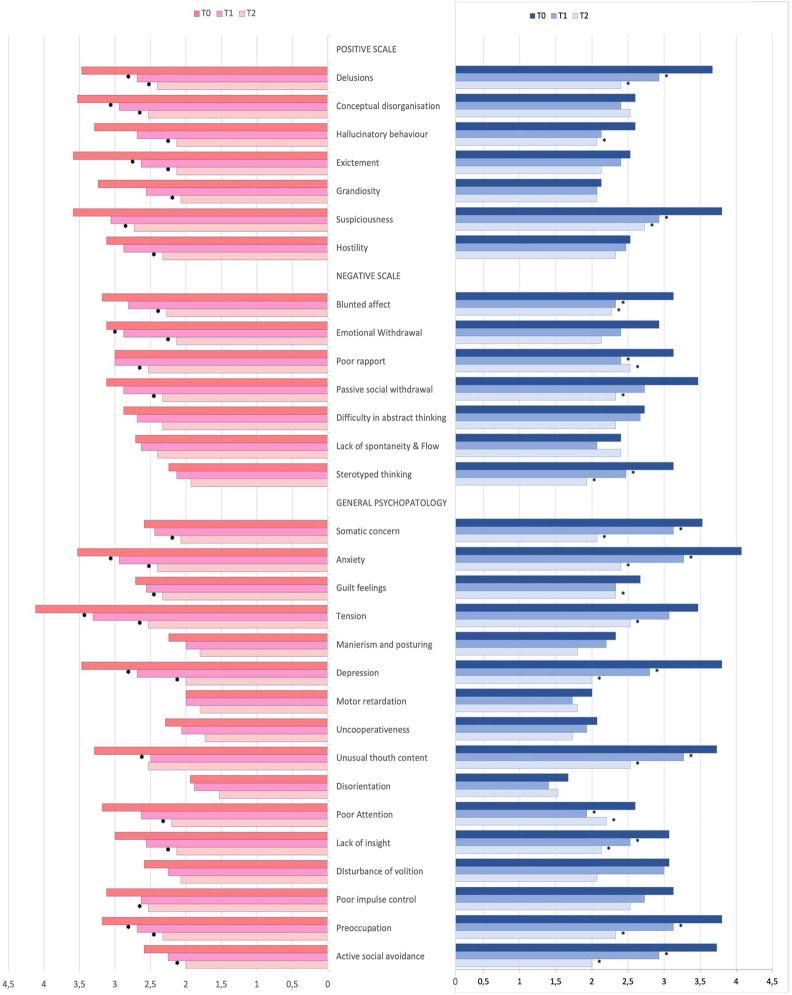
Gender-specific profiles of PANSS item scores across T0 (baseline), T1 (6-month follow-up), and T2 (12-month follow-up). *p < 0.05.

Focusing on the single items of the PANSS subscales between T0 and T1 (**Figure 1**) in men, a statistically significant decrease was found in two of the PANSS Positive symptoms scale item scores (Delusions and Suspiciousness), as well as in three of the PANSS Negative symptoms scale item scores (Blunted affect, Poor rapport, Stereotyped thinking) and eight of the PANSS General psychopathology scale (Somatic concern, Anxiety, Depression, Unusual thought content, Poor attention, Lack of insight, Preoccupation, Active social avoidance). During the same observation time (T0–T1, 6 months), notably, different results emerged in the female sample, where we found a statistically significant decrease from T0 in three out of seven of the PANSS Positive symptoms scale item scores (Delusions, Conceptual disorganization, Excitement), in just one of the PANSS Negative symptoms scale item mean scores (Emotional withdrawal), and in five of the PANSS General psychopathology scale item mean scores (Anxiety, Tension, Depression, Unusual thought content, and Preoccupation).

Moreover, we observed progressive improvements in symptom control in the T2 follow-up (**Figure 1**), especially in women. Particularly, comparing data reported in the female subsample at 12 months of follow-up (T2) with the data at baseline (T0), we found a statistically significant improvement in every single PANSS Positive symptoms scale items (Delusions, Conceptual disorganization, Hallucinatory behavior, Excitement, Grandiosity, Suspiciousness, Hostility), in four items of the PANSS Negative symptoms scale items (Blunted affect, Emotional withdrawal, Poor rapport, Passive social withdrawal), and in more than half of the PANSS General psychopathology scale item mean scores (Somatic concern, Anxiety, Guilt feelings, Tension, Depression, Poor attention, Lack of insight, Poor impulse control, Preoccupation, Active social avoidance). As for the male subsample between T0 and T2, we found a statistically significant improvement in three PANSS Positive symptoms scale items (Delusion, Hallucinatory behavior, Suspiciousness), in four PANSS Negative symptoms scale items (Blunted affect, Poor rapport, Passive social withdrawal, Stereotyped thinking), and 10 PANSS General psychopathology scale item mean scores (Somatic concern, Anxiety, Guilt feelings, Tension, Depression, Unusual tough content, Poor attention, Lack of insight, Preoccupation, Active social avoidance).

## 4 Discussion

In this naturalistic 12-month longitudinal study, we explored gender-specific outcomes on cariprazine treatment. The results show noteworthy improvements of the various dimensions of psychotic symptoms, with statistically significant and progressive reduction in all PANSS domain and Marder factor mean scores through all time points in both sexes, with the only exception of the Uncontrolled hostility/excitement PANSS Marder factor in men at both T1 and T2 with respect to T0. Furthermore, gender-specific response profiles emerged after 6 and 12 months of treatment in the PANSS symptoms between men and women.

At baseline of our observation period, we noticed a higher burden of positive symptoms of schizophrenia in women as reflected by a numerically higher mean score in the Positive symptom subscale in women. This finding is of particular interest because it is in line with previous studies, although literature doesn't always demonstrate an equally clear prevalence of positive symptoms among women (1, 26–28). Moreover, our study sample exhibited a comparable expression of negative symptoms in both women and men, contrary to the evidence that negative symptoms are often more pronounced in men (29, 30).

After 6 months of treatment (T1), we observed a statistically significant improvement across all symptom subscales in both sexes, with women showing a higher statistically significant difference in the Positive symptoms subscale. Improvements in all PANSS subscales reported at T1 widened at T2 in both sexes. It is interesting to note that the only symptom domain in which statistically significant improvement was not reported was the Uncontrolled hostility/excitement PANSS-derived Marder factor in men both at T1 and T2 (31). Exploring these data, we may argue that despite not being statistically significantly different, scores in this domain emerged to be lower among men than among women at baseline while reaching comparable scores after 6 and 12 months of treatment. This difference at baseline may account for the non-statistically significant difference. Analyzing single PANSS subscale items, we observed that cariprazine showed statistically significant effectiveness at T1 in the reduction of Excitement and Conceptual dysorganization in women, Suspiciousness in men, and Delusion in both sexes; regarding negative symptomatology, we did not observe a noteworthy improvement in women at this time point, while Blunted affect, Poor rapport, and Stereotyped thinking were the more notable improvements among the items that showed a statistically significant reduction in mean scores in men. It is worth noting that among the General psychopathology items that significantly improved, Depression, Anxiety, Unusual thought content and Preoccupation showed a significant decrease in both sexes at T1. Tension in women and Somatic concerns, Poor attention, Lack of insight, Active social avoidance in men also show significant improvements at T1.

Our data show that, with the exception of Unusual thought content in women, all PANSS items that improved at T1 continued to improve at T2, together with several additional items that reached statistical significance after 12 months compared to baseline.

Numerical reductions across all PANSS subscales were observed in both sexes at T2. Improvements in all subscales reached statistical significance in both men and women at this time point. Statistically significant improvements at T2 were confirmed in the PANSS-derived Marder factors apart from Uncontrolled hostility/excitement in men.

Most of the PANSS items showed a significant decrease in both sexes at T2 (21 items in women and 17 items in men), with greater improvements in the female subgroup. Notably, Excitement displayed an additional significant reduction not present at T1 in the Hallucinatory behaviour item which only reached significance at T2 for both men and women. Among the negative symptoms, Passive social withdrawl emerged at T2 only in both genders. Furthermore, Blunted affect and Poor rapport reached statistically significance in females at T2 only. Moreover, Guilt feelings, Somatic concern, Poor attention, Lack of insight, Poor impulse control and Active social avoidance reached statistically significant improvement in women with prolonged treatment. In men, Guilt feelings and Tension where the ones to reach statistically significant improvement with prolonged treatment with a significant reduction at T2. Comparable to what we observed in the female subgroup, Depression, Anxiety and Preoccupation emerged as the items with progressive, constant and statistically significant improvements at each time point.

Gender-specific profiles can be highlighted when comparing the improvement in each PANSS Positive symptoms subscale item score. Particularly, we found that Excitement, Conceptual disorganization, and Delusion mean scores showed a statistically significant decrease after just 6 months in women. Interestingly, a statistically significant improvement in all items scores in the PANSS Positive symptoms subscale was found after 12 months in women, demonstrating a broad symptomatic response in the female gender during prolonged treatment with cariprazine, in line with the evidence suggesting the efficacy of prolonged treatments in psychotic patients (32). Conversely, only two (delusion and suspiciousness) and three (delusion, suspiciousness, and hallucinatory behavior) item scores of the PANSS Positive symptoms subscale demonstrated a significant improvement in men after 6 and 12 months, respectively. Since comparable mean doses of cariprazine were used in men and women, we could conclude that positive symptom control was more widely achieved in women than in men treated with the same dose of the drug, suggesting a better response to antipsychotic treatment in women at this dose (14, 16). A possible explanation for the greater response to the antipsychotic therapy in women could lie in the fact that neuroleptics act more effectively on positive symptoms. In addition, the difference in drug metabolism given by differences in cytochrome systems between women and men, besides genetic differences in COMT, MAO, GABA, and DOPA systems in women and men, could also be responsible for the different responses to medications (33, 34).

As illustrated before, while the treatment of negative symptoms remains a challenging aspect in the management of schizophrenia, our analysis contributes to the growing body of literature that is exploring the efficacy of cariprazine on such symptoms, especially in men.

Our data show a growing efficacy of cariprazine on negative symptomatology through time points. Efficacy on negative symptoms, which are typically more challenging to address than positive symptoms through antipsychotic treatment and are frequently associated with poorer functioning and worse outcomes (35, 36), could be particularly beneficial for patients who still have this somewhat residual symptomatology after the resolution or weakening of positive symptoms. While the treatment of negative symptoms remains a challenging aspect in the management of schizophrenia, our analysis contributes to the growing body of literature that is exploring the efficacy of cariprazine on such symptoms. In this context, an observational study conducted by Rancans et al. (37) observed a general improvement in symptom severity in patients exhibiting symptoms. Another work by Dragasek (38) corroborated this effect on negative symptoms as reported by both patients and doctors. Furthermore, this study highlighted some practical consequences of such improvements, including enhanced work capabilities. Additionally, a randomized controlled trial (RCT) demonstrated that cariprazine monotherapy exhibited superior efficacy compared to risperidone in patients with predominant negative symptoms (39), indicating the efficacy of cariprazine in controlling such symptoms (40). This capability may be attributed to the peculiar pharmacological characteristics of cariprazine and its ability to bind to the D3 receptors, which is linked to an increase in dopaminergic tone in the prefrontal cortex (21), making it particularly effective in cases where negative symptoms constitute a significant impairment factor for the patient (41, 42).

Statistically significant improvement was also reported in all PANSS-derived Marder factor mean scores at both T1 and T2 for both genders, except for the uncontrolled hostility/excitement factor that improved significantly only in the female subsample, in line with previous results (40).

The efficacy on anxiety and depression symptoms in women is noteworthy due to the higher prevalence of these symptoms in women and the higher use of antidepressants and sedatives in women affected by schizophrenia with respect to men (5, 28, 43).

This study has some potential limitations: First, the small sample size limits the generalizability of the results; second, we did not gather information about hormonal phases or hormone-related side effects in the female subgroup, which deserve further investigation; third, a possible limitation of the present study is also the lack of information on extrapyramidal or other neuroleptic-induced neurological symptoms. However, it is important to note that this was not among the primary aims of the present study as the data on cariprazine tolerability by means of the St. Hans scale were reported in a previous paper on the 6-month follow-up data on a subsample of the present one and good tolerability outcomes were reported (22). Fourth, we did not control our analysis for substance use reported by our sample, which could be a confounding factor; this was mainly due to the small sample size. Nevertheless, given the influence of substance use disorder (SUD) on clinical presentation, such as positive and negative symptoms, and treatment outcomes, recent evidence highlighted the role of cariprazine in patients with co-occurring SUD (44, 45); this aspect deserves further examination in subsequent studies.

In conclusion, our study highlighted the difference in response to cariprazine between the two sexes, emphasizing a satisfactory antipsychotic response in women and a promising efficacy in men. Cariprazine exhibited remarkable and progressive improvements in positive symptoms in women and a distinctive capability to better control negative symptoms, as well as anxiety and depressive symptoms. Additional longitudinal studies and independent investigations are needed to further examine the efficacy profile and long-term outcomes of cariprazine treatment.

## Data Availability

The raw data supporting the conclusions of this article will be made available by the authors, without undue reservation.
